# Supplementary material for: An Assessment of Dietary Intake, Feeding Practices, Growth, and Swallowing Function in Young Children with Late-Onset Pompe Disease: A Framework for Developing Nutrition Guidelines
Source: Nutrients. 2025 Jun 1;17(11):1909. doi: 10.3390/nu17111909 (PMC12157908; doi:10.3390/nu17111909)
Supplement: Supplementary file 1 [file nutrients-17-01909-s001.zip › LOPD children nutrition study supplement table.pdf]

Supplementary Table 1. Spearman's correlations between macronutrient and caloric intake and clinical chemistry levels in children with LOPD diagnosed by newborn screening.

Shaded cells indicate a p value <0.05. Abbreviations: BUN, blood urea nitrogen; CHO, carbohydrate; CK, creatine kinase; CN, creatinine; Glc4, glucose tetrasaccharide; pro = protein.

	**calories/kg**	**protein/kg**	**% CHO**	**% protein**	**% fat**	**CK**	**BUN/CN**	**Glc4**
**calories/kg**		0.642	0.089	-0.239	-0.039	-0.068	0.277	0.266
**protein/kg**	0.642		-0.356	0.457	0.027	-0.208	0.318	0.034
**%carb**	0.089	-0.356		-0.602	-0.780	-0.024	-0.060	0.229
**%protein**	-0.239	0.457	-0.602		0.124	-0.116	0.395	-0.086
**%fat**	-0.039	0.027	-0.780	0.124		0.153	0.021	-0.284
**CK**	-0.068	-0.208	-0.024	-0.116	0.153		0.033	0.375
**BUN/CN**	0.277	0.318	-0.060	0.395	0.021	0.033		0.196
**Glc4**	0.266	0.034	0.229	-0.086	-0.284	0.375	0.196	
