# Supplementary material for: An Assessment of Dietary Intake, Feeding Practices, Growth, and Swallowing Function in Young Children with Late-Onset Pompe Disease: A Framework for Developing Nutrition Guidelines
Source: Nutrients. 2025 Jun 1;17(11):1909. doi: 10.3390/nu17111909 (PMC12157908; doi:10.3390/nu17111909)
Supplement: Supplementary file 1 [file nutrients-17-01909-s001.zip › LOPD Nutrition Questionnaire.pdf]

Duke University
Division of Medical Genetics
Late Onset Pompe Disease Nutritional Intake form

Date:

Name:

DOB:

Age: yrs mo. **Body weight:** lbs/kg **Height:** ft inches/ cm

DIAGNOSIS MADE:

Via NBS

Clinically diagnosed at age: ___ yrs ___ months

FOLLOWING SECTION NEEDS TO BE COMPLETED ONLY ONCE. IF YOU HAVE ALREADY COMPLETED IT, PLEASE SKIP TO SECTION II (PRESENT DIET)

I. INFORMATION ON DIET DURING EARLY YEARS:

A. As an infant, child was:
   1. Totally on breast milk:
   2. Totally on infant formula:
   3. Partially on breast milk and infant formula:

B. If child is/was ever given infant formula (full or partial):
   1. Age infant formula was introduced: months
   2. Name (s) of infant formula:
   3. Maximum amount of formula consumed in a day: ____ounces/ ______mL

C. Is child still drinking breast milk/infant formula? YES/NO

D. If NO, age breast milk/infant formula was stopped: __________months

E. Child is now drinking: check all that apply
   a. Dairy milk: amount consumed/day: _______ounces\mL
   b. Non-dairy milk: please name
      i. _________________ amount consumed/day: _______ounces\mL
      ii. _________________ amount consumed/day: _______ounces\mL

F. INTRODUCTION OF SOLID FOODS:
1. Was a swallow evaluation done prior to introduction of solids? Y/N
2. Age at solids introduction:
3. If beyond six months, delay in solids introduction was due to:
   a. Difficulty with eating solids (chewing, swallowing etc):

b. Refusal to eat solids:
c. Other reasons (please mention):

G. REGARDING APPROACH TO DIET DURING INFANCY, TODDLER AND PRE-SCHOOL YEARS:

	**Infancy (< 1 year)**	**Toddler (1-3 years)**	**Pre-school and later**
**FOOD GROUPS ENCOURAGED** (eg. Starch, meats, vegetables, fruits, dairy, fats etc)			
**FOOD GROUPS AVOIDED OR DISCOURAGED:**			

Above diet was implemented because of (all that apply)

a. Consultation with a dietitian/physician
b. Your nutritional knowledge/beliefs
c. Internet research
d. Help of social media/support group
e. Advice from family and friends

H: Any additional information/comments you want to share regarding your child's early nutrition:

II. PRESENT DIET:

1. Is your child on a special diet for Pompe disease now: YES/NO

If on "special diet":

Foods/food groups restricted:

Foods/food groups encouraged:

2. Was above recommended or implemented under the guidance of a dietitian? YES/NO

Continued on next page: Food intake

3. FOOD INTAKE ON A TYPICAL DAY AT THE PRESENT TIME

For TIME: Please record food intake at all the meals (breakfast, lunch, dinner) and snacks.

For DESCRIPTION OF FOOD ITEMS: note homemade (with list of major ingredients used) vs purchased from restaurant. List the brand name of the food where available.

For AMOUNT EATEN: for liquids note in ounces or cup measures. For solids note weight in ounces or grams, cup measures, TBS, Tsp, # of pieces.

TIME	DESCRIPTION OF FOOD ITEMS	AMT EATEN	CARB	FAT	PRO	CALORIES
			FOR DIETITIAN USE			

TIME	DESCRIPTION OF FOOD ITEMS	AMT EATEN	CARB	FAT	PRO	CALORIES
			FOR DIETITIAN USE			

Supplements (multivitamin, Vit D, calcium, iron, fiber, probiotic, protein etc):
____

TOTAL SERVINGS FROM EACH FOOD GROUP CONSUMED IN A DAY (FROM FOOD LOG ABOVE):

(TO BE COMPLETED WITH DIETITIAN)

FOOD GROUPS	OUNCES/GM	TBS/TSP/CUP	# OF PIECES	TOTAL
STARCH				
MEAT/PROTEIN				
MILK				
VEGETABLE				
FRUIT/JUICE				
FAT				
BEVERAGES:				
Water				
Other				

Any additional information/comments you want to share regarding your child's present nutrition:

